# Mechanistic insights into photochromic 3*H*-naphthopyran showing strong photocoloration

**DOI:** 10.1038/s41598-022-14679-9

**Published:** 2022-06-24

**Authors:** Błażej Gierczyk, Michał F. Rode, Gotard Burdzinski

**Affiliations:** 1 Faculty of Chemistry, Adam Mickiewicz University in Poznań, Uniwersytetu Poznańskiego 8, 61-614 Poznań, Poland; 2 Institute of Physics, Polish Academy of Sciences, Aleja Lotników 32/46, 02-668 Warsaw, Poland; 3 Faculty of Physics, Adam Mickiewicz University in Poznań, Uniwersytetu Poznańskiego 2, 61-614 Poznań, Poland

**Keywords:** Photochemistry, Physical chemistry, Optical materials

## Abstract

3,3-Diphenylbenzo[*f*]chromene (**1**) represents an important architectural platform for photochromic systems. Since the practical utility of such chromophores is largely dependent upon the kinetics of coloration and decoloration, elucidating the mechanistic details of these processes is of great value. Toward this end, we studied the photochromic reaction of (3-(2-methoxyphenyl)-3-phenyl-3*H*-benzo[*f*]chromene (**2**) by both time-resolved UV–vis and mid-IR spectroscopies. We found that irradiation of **2** at 365 nm generates long-lived colored *transoid-cis* isomers with lifetimes of 17.1 s and 17.5 min (at 21 °C) and even longer-lived *transoid-trans* isomers with a lifetime of 16 h. These experimental results were supplemented with ab initio ground-state and excited-state calculations, and the resulting theoretical interpretation may be useful for the design of new photochromic systems with optimized photofunctionality.

## Introduction

**Figure 1:**
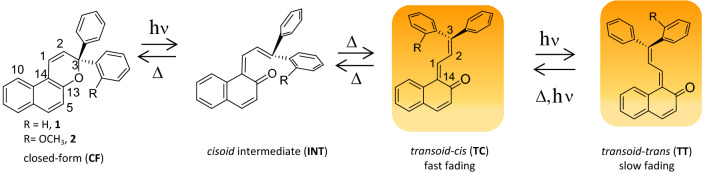
UV excitation of 3,3-diphenyl-3*H*-naphtho[2,1-*b*]pyran (**1**) converts the closed form **CF** to color isomers **TC** (short-lived) and **TT** (long-lived), with the intermediacy of *cisoid-cis* form **INT** (ultrashort-lived)^11^. Compound **2** with a methoxy group implemented at one of the phenyl rings at *ortho* position is selected for studies of a strong photocoloration effect.

Among the known photochromic compounds, the family of 3,3-diphenyl-3*H*-naphtho[2,1-*b*]pyrans (**1**) has attracted great interest in the field of ophthalmic lens production^1–4^. Compound **1**, exhibiting both P and T type photochromism, has been the subject of fundamental research^5–13^. In the photochromic reaction under typical conditions two colored species are formed (Fig. 1): *transoid*-*cis* **TC** (which fades rapidly) and *transoid*-*trans* **TT** (which fades more slowly)^5^. Synthetic changes in the structural pattern of **1** can tune its properties to fulfil the requirements of a particular application.

When the application requires a relatively fast decoloration rate, the presence of the long-lived **TT** form is unwanted. Efforts are focused then on designing derivatives of **1** with a minimized **TT** contribution in the photoreaction. Under typical experimental conditions with UV irradiation, the active channel for **TT** generation is **TC** → **TT** photoisomerization, which is a *single-twist* long amplitude motion^11,13^ occurring in the excited state (S_1_). Strong competition of the other **TC** excited state deactivation channels can minimize the **TC** → **TT** photoisomerization yield. For example, if the potential**-**energy landscape of the initially photoexcited **TC** S_1_ state favors a *bicycle-pedal* motion, the isomerization path might be quickly aborted through S_1_ → S_0_ internal conversion populating the form with the geometry close to that of the starting **TC** form^12,13^. Such a deactivation path can reduce the **TC** → **TT** photoisomerization yield, as reported for derivative of **1** with a methoxy group inserted at the position 10^13,14^.

**Figure 2:**
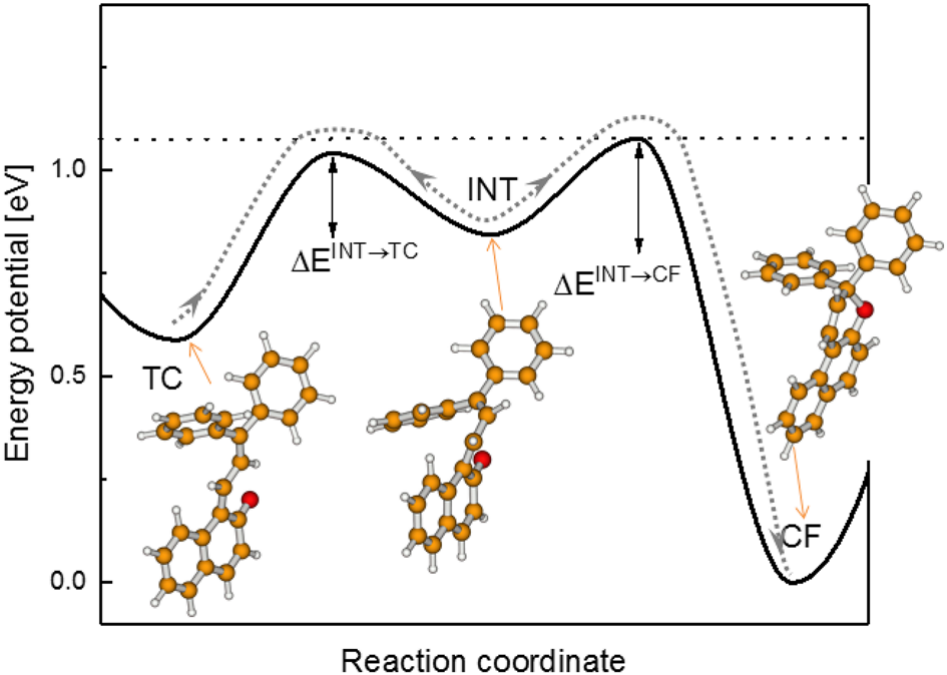
Thermal decoloration of **TC** → **CF** for compound **1** involves two reaction steps **TC** → **INT** and **INT** → **CF**, the first step is reversible^15^.

The thermal fading rate of colored **TC** and **TT** isomers is also a key parameter of photochromic materials based on derivatives of **1**. Appropriate substituents in the skeleton of **1** can be used to tune the **TC** color fading rate which depends on the ground state potential energy landscape^15^. A key role in this process is played by the *cisoid-cis* intermediate form (**INT**) lying midway between the colored **TC** form (see Fig. 2), and the colorless **CF** form^15^. The relationship between the energy barriers ΔE^INT-TC^ and ΔE^INT-CF^ separating the intermediate **INT** from nearby global and local minima may either decelerate the **INT** → **CF** process (favoring the return to the colored **TC** form) or accelerate the process (favoring the ring-closing reaction). For instance, the ΔE^INT-TC^ barrier for **1** is lower than ΔE^INT-CF^ (Fig. 2), which makes the apparent **TC** lifetime relatively long (9.3 s at 21 °C in cyclohexane).

The opposite situation (ΔE^INT-TC^ > ΔE^INT-CF^) has been reported for 3*H*-naphthopyrans with phenyl substitution at position **2**^15^. The apparent **TC** lifetime is remarkably short (only 30 μs) in solution at room temperature^16^. On the other hand, one can expect that a slow decoloration would help to achieve a strong photocoloration effect in the photostationary state. It is widely accepted that inclusion of a bulky substituent at the *ortho* position of one of the phenyl rings in **1** would result in a substantial extension of the thermal fading half-life ${\tau }_{1/2}$^2,4,17–21^. The half-life ${\tau }_{1/2}$ of the open forms gets longer with increasing size of the *ortho* substituent (in the order of H → F → MeO → Me → Cl → Br → I) in the phenyl ring of 3-aryl-3-(4-pyrrolidinophenyl)naphtho[2,1-*b*]pyrans^22^. A remarkably slow-decoloration rate remains an intriguing feature for mechanistic and spectroscopic investigations.

For our studies we selected 3-(2-methoxyphenyl)-3-phenyl-3*H*-benzo[*f*]chromene (**2**), Fig. 1, as a candidate for a material with the strongest UV activated coloration. The relatively simple structure of **2** facilitates ab initio quantum-chemical calculations for the excited state, while the stages of the photochromic reaction can be studied in detail with experimental techniques such as the time-resolved UV–vis and mid-IR spectroscopies.

## Experimental

### Materials

Compound **1** was purchased from TCI. Compound **2** was synthesized following the procedures described in the Supplementary Information. In the time-resolved spectroscopic investigation cyclohexane of spectroscopic grade from Sigma Aldrich was used for solution preparation.

### Time-resolved UV–vis and mid-IR spectroscopies

Changes in UV–vis absorption spectra and kinetics were recorded using three configurations.

1. Jasco V-550 spectrophotometer with a modified cell compartment. The solution in 1 cm × 1 cm fused silica cuvette was placed into a temperature-controlled cuvette holder (Flash 300, Quantum Northwest) with stirring switched on. UV LED (λ_exc_ = 365 nm, Thorlabs M365LP1) was used to induce the photochromic reaction (as in the two other configurations below).
2. Similar arrangement with a temperature-controlled cuvette holder and white light generated by a xenon lamp (Applied Photophysics), equipped with a bundle fiber, as a probing beam. The probing beam was passed through an almost-closed iris to ensure low white light intensity. The UV–vis spectra were recorded by an Ocean Optics FLAME-T-VIS–NIR-ES USB spectrometer at the sampling rate of 10 spectra per second.
3. Acquisition mid-IR spectra upon simultaneous measurements in UV–vis spectral range has been described recently^11^. A Bruker Tensor 27 FT-IR spectrometer was equipped with an MCT detector, with a spectral resolution of 4 cm^−1^ and a sampling rate of 1.15 s^−1^. The UV–vis probing light was generated from the xenon lamp. An Ocean Optics spectrophotometer was used for recording of UV–vis spectra. A Harrick Scientific cell was used with 2 mm thick CaF_2_ window and a 0.63 mm spacer.

### Theoretical calculations

The equilibrium geometries of the **CF** conformers and their isomers in the closed-shell ground state (S_0_) were obtained with the MP2 method^23^ with no imposed symmetry constrains. The energy of the most stable form **CF**_**c**_ (Table S1, Supplementary Information) was assumed as the reference energy for higher energy structures. The excited-state (S_1_) equilibrium geometries were determined with the second-order algebraic diagrammatic construction ADC(2) method^24–26^. The correlation-consistent valence double zeta basis set with polarization functions on all atoms (cc-pVDZ)^27^ was used in these calculations as well as in the potential energy profiles and surfaces. The vertical excitation energies and response properties of the lowest singlet excited states were calculated using the CC2 methods^28,29^. The basis set augmented with the diffuse functions aug-cc-pVDZ was also used to compute vertical excitation energies of the molecular system. All calculations were performed using the TURBOMOLE program package^30^.

## Results and discussion

The stationary UV–vis absorption spectrum of **2** in cyclohexane (Figure S1, Supplementary Information) is similar to that of the reference compound **1**^11^, reflecting structural similarities between the two derivatives. In the structure of **1**, each phenyl ring has two *ortho* positions that can be substituted by a methoxy group, thus, four respective conformers of **2** are taken into account in the calculations. These conformers of the **CF** form are in thermal equilibrium in freshly prepared solution (indicated with subscripts: a, b, c, and d, see Table S1). The selection of wavelength at 365 nm induces the electronic transition S_0_ → S_1_(π,π*) separately in each **CF** form, which opens up along the C_3_–O_4_ photodissociation pathway, as in **1**^11^. The photoinduced pyran ring-opening process can lead to the colored-isomers **CTC** and **TTC**. All the **CF**, **TC** and **TT** conformers potentially involved in the photoreaction, along with their calculated vertical excitation energies (ΔE^VE^) simulating UV–vis absorption spectra, are collected in Table S1. The strong S_0_ → S_2_(π, π*) transitions for colored **TC** forms are slightly redshifted *vs.* those of **TT** forms; a similar trend was observed for the parent compound **1**^11^.

**Figure 3:**
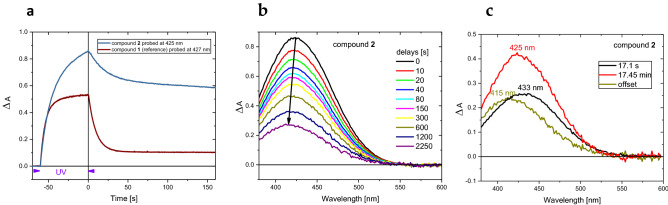
(**a**) Kinetics of the changes in absorption as a result of UV irradiation (4.1 mW/cm^2^) in the period of 60 s for **2** and the reference **1** in cyclohexane at 21 °C (both solutions prepared with the same absorbance A(365 nm, 1 cm) = 0.24). (**b**) Transient UV–vis absorption spectra for **2** after switching off UV irradiation. (**c**) Decay associated spectra of the **TC** (17.1 s and 17.5 min) and **TT** isomers (the offset, long-lived population) obtained by global analysis using a biexponential function.

To study the photochromic reaction by changes in UV–vis absorption spectra, a UV LED light at 365 nm was used for sample excitation, while a xenon lamp was used for probing. Figure 3a shows the kinetics probed at 425 nm upon 60-s exposure a solution of **2** in cyclohexane to UV light.

Compared to **1**, compound **2** produces a stronger absorption signal upon UV irradiation (Fig. 3a), and after cessation of UV irradiation its color fading occurs at a lower rate. The quantum yield of **TC** formation is similar for **2** and **1** (0.745^11^), since the early signal rise in the − 55 to − 60 s time window shows the same slope and the **TC** molar extinction coefficients are comparable (see Supplementary Information). Figure 3b shows the evolution of the transient UV–vis absorption spectra in the time window of 0–2250 s. Global analysis of data (Fig. 3c) reveals three distinct populations: **TC1** with the absorption band peaking at 433 nm and a lifetime of 17.1 s, **TC2** with the band maximum at 425 nm and a 17.5 min lifetime, and **TT** with a maximum at 415 nm and a long lifetime. The **TT** lifetime of 16 h was determined in an additional experiment performed in the time window of 40 h (Figure S2). The respective molar extinction coefficients, ${\varepsilon }_{max}(TC1)$ ≈ 18,900 M^−1^ cm^−1^, ${\varepsilon }_{max}(TC2)$ ≈ 18,700 M^−1^ cm^−1^ and ${\varepsilon }_{max}(TT)$ ≈ 29,500 M^−1^ cm^−1^, are deduced from the simultaneously recorded changes in the UV–vis and mid-IR spectral ranges (see Figure S3, Supplementary Information). Thus, one can estimate from the data shown in Fig. 3 that at the moment of UV irradiation cessation, the conversion of **CF** population to colored forms is 69% and the concentrations of [**TC1**], [**TC2**] and [**TT**] are: 1.4 × 10^–5^ M, 2.2 × 10^–5^ M and 0.8 × 10^–5^ M, respectively. In addition, the FT-IR spectroscopy provides a confirmation of our signal assignment to **TC** and **TT** isomers, since the C=O stretching absorption band of **TC** is located at a lower frequency than that of **TT**, as has been already observed for the reference compound **1** (**TC** at 1644 cm^−1^ and **TT** at 1655 cm^−1^^11^).

**Figure 4:**
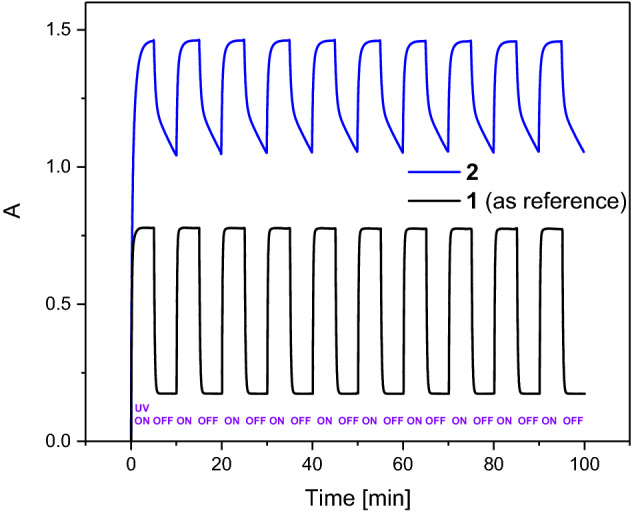
Kinetics of absorption upon switching on and off UV light at 365 nm (4.1 mW/cm^2^) for **2** and **1** in cyclohexane probed at 430 nm using a Jasco spectrophotometer, temperature set at 21 °C. Solutions prepared with the same absorbance A(365 nm, 1 cm) = 0.39.

Photocycle reproducibility observed for **2** in cyclohexane is similar to that for **1**, which is known to have high fatigue resistance (Fig. 4).

### Why do TC forms differ in decoloration kinetics?

The **TC** form in 3*H*-naphthopyrans is photogenerated in a single-photon absorption process^11^. A single **TC** isomer is generated from compound **1**, while four **TC** isomers from compound **2** should be taken into consideration. The geometries of these conformers are presented in the first column in Table S1. **CTC**_**a**_ and **CTC**_**b**_ are energetically more stable than **TTC**_**c**_ and **TTC**_**d**_**.** Each of the four **TC** isomers is a colored species which, under thermal conditions, fades towards the respective closed **CF** form via a single *cisoid-cis* intermediate **INT** geometry located on the ground-state potential energy pathway. Each of the four S_0_-state pathways is characterized by a slightly different energy barriers separating the intermediate **INT** structure from the local **TC** and **CF** minima, which influence the apparent fading rate of each **TC** isomer. Energy barriers are shown in Table 1.

**Table 1:** Energetics of the two-stepwise process of the **TC** form depopulation (**TC** ↔ **INT** → **CF**). Adiabatic S_0_-state energies, *E*^*a*^ (in eV), and dipole moment, μ_*g*_ (in Debye)**,** for the relevant minima: **TC**, **INT**, **CF**, and transition states separating these minima: **TS1** and **TS2**, calculated at the MP2/cc-pVDZ theory level.

Form		∆E^TC-out^	**TS2**	∆E ^INT-TC^	**INT**	∆E^INT-CF^	**TS1**	**CF**
**CTC ↔ INT → CF**, τ(CTC) = 17.5 min in cyclohexane at 21 °C								
**CTC**_**a**_								
	0.626 eV 3.51 D	+ *0.380*	1.005 4.02	+ *0.161*	0.844 1.25	+ *0.341*	1.185 1.22	0.056 1.79
**CTC**_**b**_								
	0.610 eV 4.11 D	+ *0.414*	1.024 3.62	+ *0.166*	0.858 2.93	+ *0.364*	1.223 2.10	0.064 2.61
**TTC ↔ INT → CF**, τ(TTC) = 17.1 s in cyclohexane at 21 °C								
**TTC**_**c**_								
	0.655 eV 3.13 D	+ *0.415*	1.070 2.17	+ *0.164*	0.906 1.79	+ *0.254*	1.160 1.90	0.00 2.37
**TTC**_**d**_								
	0.669 eV 2.17 D	+ *0.476*	1.145 1.76	+ *0.207*	0.938 1.53	+ *0.203*	1.141 1.90	0.113 1.61

The first reaction step in the **TC** fading process is **TC** → **INT** isomerization, which must overcome a relatively high energy barrier ΔE^TC-OUT^ (~ 0.38−0.48 eV), to reach a highly energetic *cisoid-cis* **INT** intermediate in an endothermic process. In the second step, this intermediate can follow (1) an exothermic reaction towards the final closed-pyran ring **CF** form or (2) an alternative reverse process towards the **TC** form (Figure S4, Supplementary Information). As one can see in Table 1, the ΔE^INT-CF^ barrier towards the final **CF** for each fading pathway is usually higher than the respective ΔE^INT-TC^ barrier towards the initial **TC** form (except for the **TTC**_**d**_ pathway, for which the **TS2**_**d**_ structure (E^a^ = 1.145 eV) is slightly more destabilized due to O…O repulsion). Such a situation (ΔE^INT-CF^ > ΔE^INT-TC^) favors deceleration of the **TC** fading process, since once **INT** is formed it would rather repopulate the **TC** form. This may explain long **TC** lifetimes observed in the experiment for **2** (17.1 s and 17.5 min) in comparison to that of **1** (9.3 s, ΔE^INT-CF^ = 0.233 eV and ΔE^INT-TC^ = 0.196 eV^13,15^).

In order to assign each of the two **TC** lifetimes (17.1 s and 17.5 min) to the respective **CTC** and **TTC** families, we should consider their depopulation following the S_0_-state energetic profile (Figure S4). While the ΔE^INT-TC^ barrier of ~  + 0.16 eV is the same for all the considered **TC** isomers (expect for **TTC**_**d**_), it is the ΔE^INT-CF^ barrier that seems to be critical. Since ΔE^INT-CF^ is higher for **CTC** than for **TTC** (~ + 0.35 *vs*. 0.25 eV), the equilibrium of **CTC**_**a**_ and **CTC**_**b**_ is responsible for the long 17.5 min time-constant. The short time-constant (17.1 s) should be then ascribed to the **TTC** forms (equilibrium of **TTC**_**c**_ and T**TC**_**d**_). The respective equilibria (**CTC**_**a**_ − **CTC**_**b**_ and **TTC**_**c**_ − **TTC**_**d**_) are confirmed by the relatively low interconversion S_0_-state energy barriers of ~  + 0.2 eV.

To support the idea that the high value of the ΔE^INT-CF^ barrier is a decisive parameter in longer fading time of **CTC** forms, we analyzed the geometries of the transition state **TS1** structures. Indeed, the presence of a methoxy group is the cause of the O…O repulsion in **TS1**_**a**_ (E^a^ = 1.185 eV, see Table 1) or 10H…OCH_3_ steric hindrance in **TS1**_**b**_ (E^a^ = 1.223 eV). The *ortho*-methoxy substituent increases the electron density on that aromatic ring, which has a stabilizing effect on the *cisoid-cis* **INT** geometry through increased π-stacking of the aryl and naphthalenone moieties. This is responsible for a higher ΔE^INT-CF^ barrier in the case of **INT**_**a**_ and **INT**_**b**_ (+0.341 eV and +0.364 eV) *vs*. **INT**_**c**_ and **INT**_**d**_ (+0.254 eV and +0.203 eV).

### Mechanism of the TC → TT photoisomerization reaction in the singlet excited state

**Figure 5:**
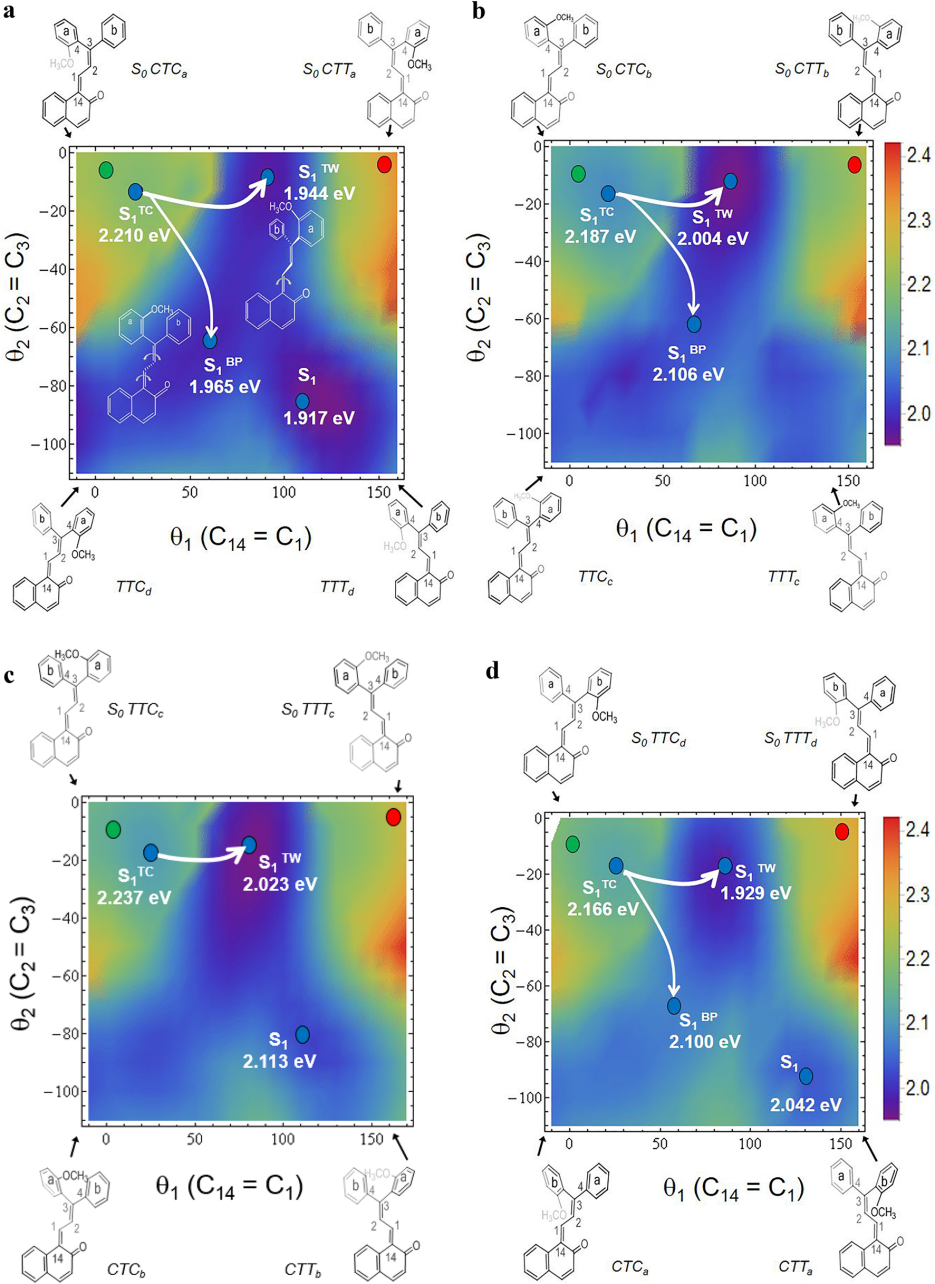
Minimum-potential-energy surface of the lowest excited electronic state of the **TC**-molecule: (**a**) **CTC**_**a**_, (**b**) **CTC**_**b**_, (**c**) **TTC**_**c**_ and (**d**) **TTC**_**d**_, plotted as a function of θ_1_(C_14_=C_1_) and θ_2_(C_2_=C_3_) torsional angle coordinates. Green circle represents the Franck–Condon region of the ground-state **S**_**0**_^**TC**^ local minimum and red circle represents the ground-state **S**_**0**_^**TT**^ local minimum. Blue circles represent various types of the excited-state minima: **S**_**1**_^**TC**^—the minimum initially populated after **S**_**0**_^**TC**^ photoexcitation, **S**_**1**_^**BP**^—achieved through the *bicycle-pedal* motion, and **S**_**1**_^**TW**^—reached by *single-twist* motion mechanism. The results were obtained with the aid of the ADC(2)/cc-pVDZ method, for the excited state, and with the MP2/cc-pVDZ, for the ground state.

The act of photon absorption by each **TC** isomer activates the two double bonds present in the C_14_=C_1_–C_2_=C_3_ bridge linking the naphthalenone skeleton with the diphenylmethylene rotor. This double bond activation allows free rotation about these bonds in the singlet excited state. As already shown for **1** derivatives^13^, this rotation can be classified as a *single-twist* mechanism (rotation around the C_14_=C_1_ bond) or as a *bicycle-pedal* motion (if the concerted rotation about both double bonds takes place). Both mechanisms are visualized in the excited-state potential energy surface spanned over the two double bonds (Fig. 5). The *single-twist* motion can be seen as a motion along the X axes (C_14_=C_1_ rotation) from the green dot representing the initial **TC** geometry (upper left corner of the contour plot) towards the red dot (**TT** form, in the upper right corner). This motion meets the excited state **S**_**1**_^**TW**^ minimum for which C_13_–C_14_=C_1_–C_2_ dihedral angle is usually a little less than 90°, but it should be considered as the active channel in **TC** → **TT** photoisomerization. Alternatively, the *bicycle-pedal* motion is followed along the diagonal of the plot—from the **TC** form (green dot, upper left corner) towards the bottom right corner. This path encounters the **S**_**1**_^**BP**^ minimum midway through, which may deactivate through S_1_ → S_0_ internal conversion to repopulate eventually the initial **TC** geometry in the S_0_ state. Inspection of the contour plots in Fig. 5 and energetics in Table S2 shows that the relative energies of **S**_**1**_^**TW**^ are below **S**_**1**_^**BP**^ thus the photoisomerization **TC** → **TT** is expected to be efficient, as observed in the experiment.

According to theoretical calculations, the replacement of the methoxy group in **2** by a methyl substituent has a low impact since its potential energy surface is also tilted towards the **S**_**1**_^**TW**^ minimum (see Table S3). Consequently, this system should also easily produce the **TT** form.

## Conclusions

The synthesized compound **2** shows exceptional properties among the members of 3*H*-naphthopyran family. In the photochromic reaction its colored isomers are formed with longer lifetimes in comparison to that of the reference compound **1**. Under UV irradiation a high accumulation of colored forms is observed in a photostationary state. The mechanism of the colored **TC** form fading process can be analyzed using the energetic landscape of the thermal **TC** → **INT** → **CF** reaction. The determined ΔE^INT-CF^ energy barrier seems to hamper **CF** repopulation and extends the apparent **TC** lifetime. Thus, the experimentally determined long lifetime of 17.5 min can be assigned to **CTC** forms, while the short lifetime of 17.1 s to **TTC** conformers. The theory suggests also that all **TC** isomers can undergo photoisomerization by the *single-twist* mechanism around the C_14_=C_1_ angle. The photogenerated **TT** form is long-lived—the population decay occurs with a single time-constant of 16 h at 21 °C. The proposed photochromic reaction mechanism explains the strong photocoloration effect observed for **2**. Theoretical investigations should be considered as an efficient tool for designing new 3*H*-naphthopyrans derivatives with optimized properties. In other words, the implementation of a promising substituent in 3,3-diphenyl-3*H*-naphtho[2,1-*b*]pyran skeleton can be first theoretically tested for desired photochromic properties prior to the synthesis efforts.

## Supplementary Information

Supplementary Information.

## Data Availability

The datasets generated during the current study are available from the corresponding author on request.
