# Supplementary material for: Mechanistic insights into photochromic 3H-naphthopyran showing strong photocoloration
Source: Sci Rep. 2022 Jun 24;12:10781. doi: 10.1038/s41598-022-14679-9 (PMC9232484; doi:10.1038/s41598-022-14679-9)
Supplement: Supplementary file 1 — Supplementary Information. [file 41598_2022_14679_MOESM1_ESM.pdf]

Supplementary data for
Mechanistic insights into photochromic 3*H*-naphthopyran showing strong photocoloration

Błażej Gierczyk[a], Michał F. Rode[b,*] and Gotard Burdzinski[c,*]

[a] Faculty of Chemistry, Adam Mickiewicz University in Poznań, Uniwersytetu Poznańskiego 8, 61-614 Poznań, Poland,
[b] Institute of Physics, Polish Academy of Sciences, Aleja Lotników 32/46, 02-668 Warsaw, Poland, E–Mail: mrode@ifpan.edu.pl (M.R.),
[c] Faculty of Physics, Adam Mickiewicz University in Poznań, Uniwersytetu Poznańskiego 2, 61-614 Poznań, Poland, E–Mail: gotardb@amu.edu.pl (G.B.)

Synthesis of compound **2**

(3-(2-Methoxyphenyl)-3-phenyl-3*H*-benzo[*f*]chromene

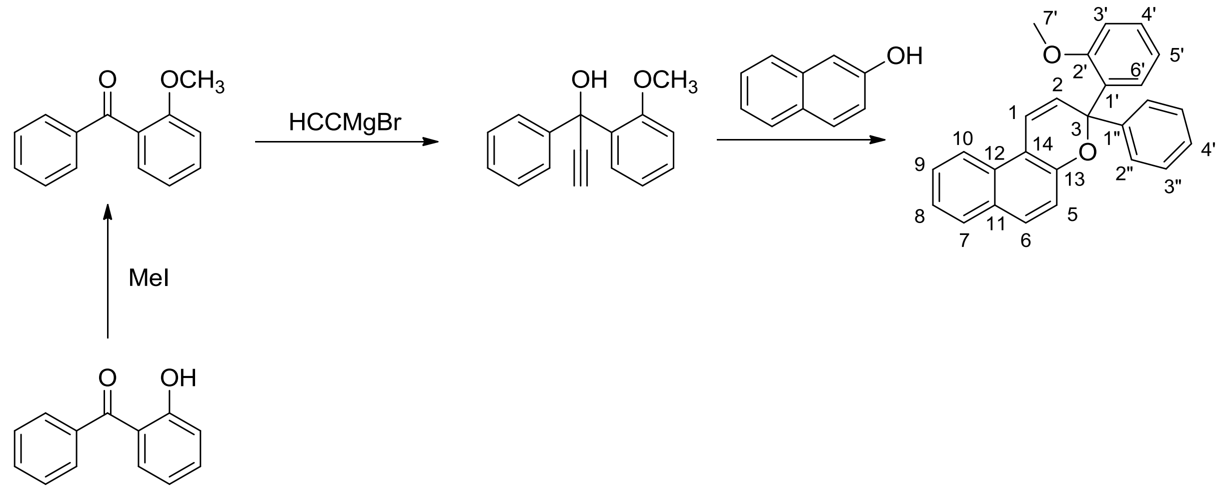

2-Methoxybenzophenone

To the solution of 2-hydroxybenzophenone (2.00 g, 10.1 mmol) in dry acetone (50 mL), anhydrous potassium phosphate (5.00 g, 23.6 mmol) and iodomethane (1.3 mL, 20.9 mmol) were added. The mixture obtained was stirred and refluxed over 5 h. After cooling, the solids were filtered off, and the solvent was evaporated. Product was purified by column chromatography on silica, using chloroform/hexane 1:1 (v/v) as eluent; yield 2.02 g (94%) as a colorless oil.

^{1}H NMR ($CDCl_3$): δ 7.81 (2H, *pseudo*d), 7.54 (1H, *pseudo*t), 7.46 (1H, dd), 7.42 (2H, *pseudo*t), 7.35 (1H, dd), 7.04 (1H, t), 6.99 (1H, d), 3.72 (3H, s).

^{13}C NMR ($CDCl_3$): δ 196.4, 157.3, 137.8, 133.0, 131.8, 129.8, 129.6, 128.9, 128.2, 120.5, 111.4, 55.6.

1-(2-Methoxyphenyl)-1-phenyl-2-propyn-1-ol

2.00 g (9.4 mmol) of 2-methoxybenzophenone was dissolved in dry THF (50 ml) and 50 ml of 0.5 M ethynylmagnesium bromide (25 mmol) solution in THF was added dropwise. The mixture obtained was stirred over 24 h at room temperature, then 100 ml of 5% aqueous NH_4Cl solution was added. The product was extracted with diethyl ether (2 x 100 ml), the organic layer was dried over Na_2SO_4, and the solvent was evaporated in a vacuum. The obtained oil was purified by flash chromatography on SiO_2, using $CHCl_3$ as eluent; yield: 1.65 g (74%) of a yellow oil.

^{1}H NMR ($CDCl_3$): δ 7.54 (2H, *pseudo*d), 7.40 (1H, dd), 7.34 (2H, *pseudo*t), 7.31 (1H, ddd), 7.29 (1H; *pseudo*t), 6.96 (1H, td); 6.93 (1H, dd), 4.90 (1H, s), 3.76 (3H, s), 2.82 (1H, s).

^{13}C NMR ($CDCl_3$): δ 156.8, 144.0, 132.1, 129.6, 128.4, 128.0, 127.6, 126.2, 120.9, 112.2, 85.6, 75.1, 74.2, 55.8.

3-(2-Methoxyphenyl)-3-phenyl-3*H*-benzo[*f*]chromene

A solution of 1.00 g of 1-(2-methoxyphenyl)-1-phenyl-2-propyn-1-ol (4.2 mmol), 0.61 g of 2-naphthol (4.2 mmol), 1.5 mL of triethyl orthoformate (9 mmol), and 50 mg of pyridine tosylate (catalyst) in 30 mL of dry 1,2-dichloroethane was heated over 5 h under reflux in an inert atmosphere and protected from light. After cooling, the solvent was evaporated in a vacuum, the residue was dissolved in 25 mL of dichloromethane, and this solution was washed with

water (2 x 20 mL). The organic layer was dried over Na_2SO_4, evaporated, and the product obtained was purified by column chromatography on SiO_2 using chloroform/hexane 1:1 (v/v) as eluent; yield 1.37 g (89%) as off-white fluffy crystals (m.p. 152-153°C).

^{1}H NMR ($CDCl_3$): δ 7.96 (1H, d, 8.3 Hz; H7), 7.76 (1H, dd, 7.7 & 1.7 Hz; H6'), 7.71 (1H, d, 8.1 Hz; H10), 7.65 (1H, d, 8.8 Hz; H6); 7.46 (1H, overlapped; H8), 7.45 (2H, *pseudo*d; H2"), 7.31 (1H, ddd, 8.1, 6.8 & 1.0 Hz; H9), 7.28 (3H, m; H4', H3"), 7.25 (1H, d, 10.2 Hz; H1), 7.22 (1H, *pseudo*t; H4"), 7.19 (1H, d, 8.8 Hz; H5), 6.97 (1H, dd, 7.7 & 1.1 Hz; H5'), 6.88 (1H, dd, 8.1 & 0.9 Hz; H3'), 6.56 (1H, d, 10.2 Hz; H2), 3.56 (3H, s; H7')

^{13}C NMR ($CDCl_3$): δ 155.7 (C2'), 150.3 (C13), 144.4 (C1"), 132.4 (C1'); 129.8 (C11), 129.4 (C6), 129.3 (C12), 128.9 (C4'), 128.5 (C10), 127.8 (C3"), 127.4 (C6'), 127.3 (C4"), 127.1 (C2"), 126.8 (C2), 126.4 (H8), 123.4 (C9), 121.3 (C7), 120.5 (C5'), 118.6 (C1), 118.3 (C5), 113.8 (C14), 112.4 (C3'), 82.1 (C3), 55.5 (C7')

2-Methoxybenzophenone in $CDCl_3$

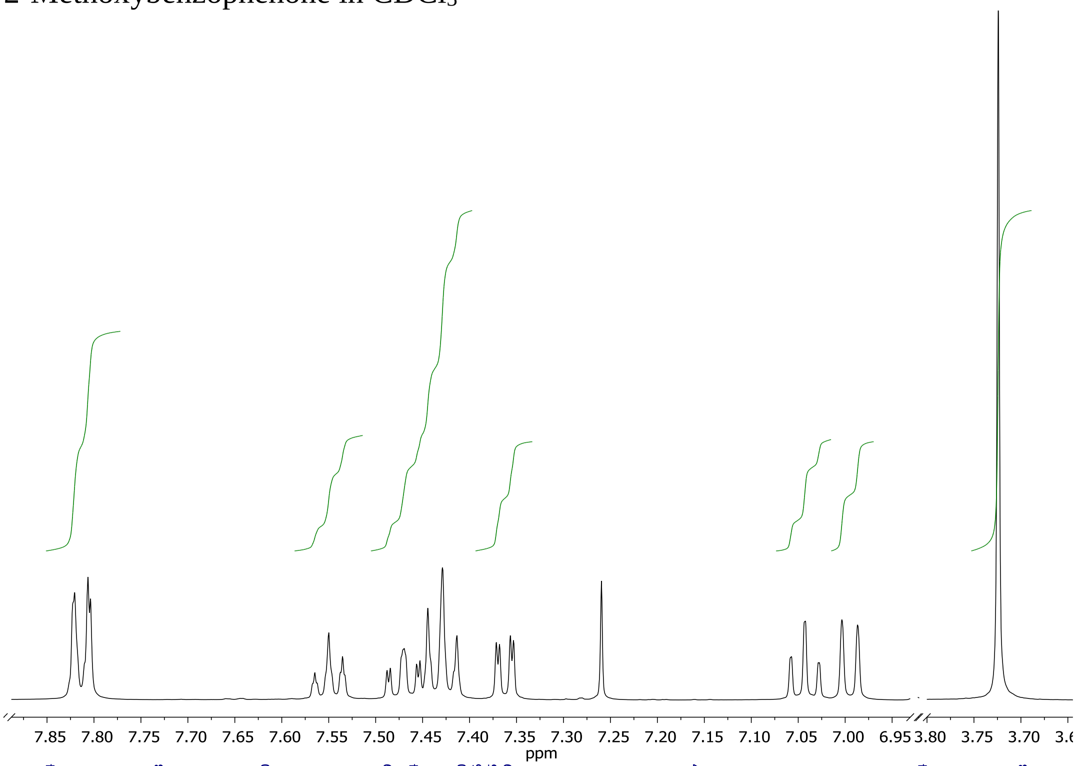

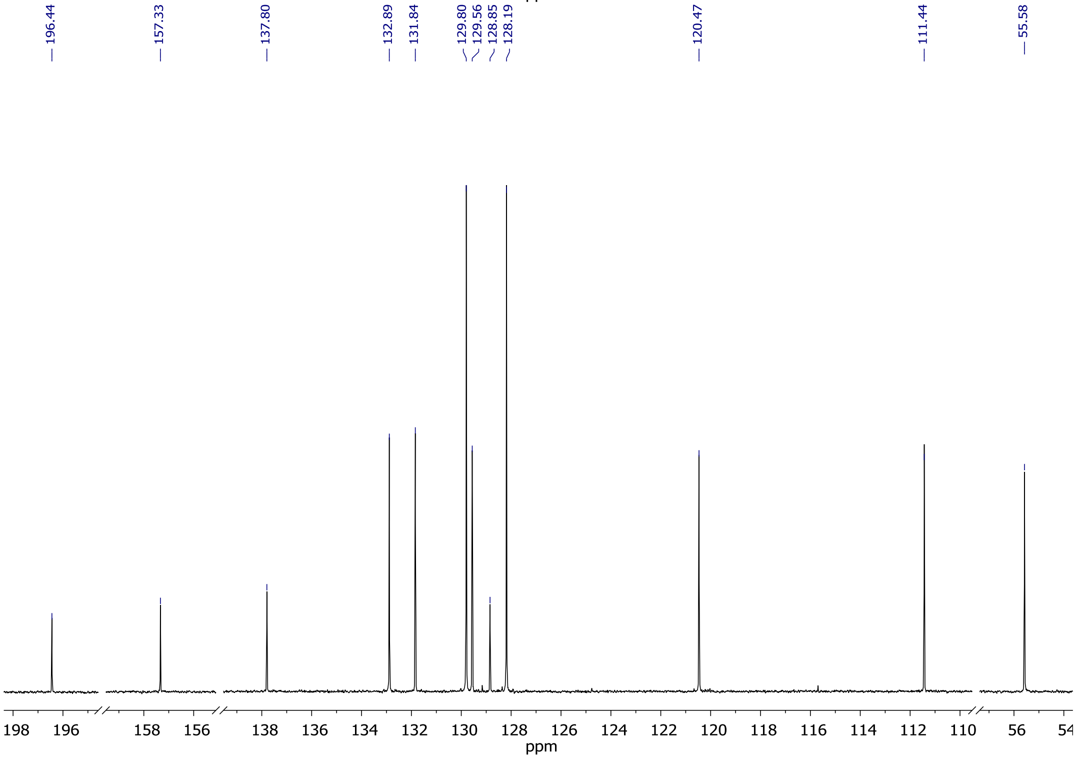

1-(2-Methoxyphenyl)-1-phenyl-2-propyn-1-ol in $CDCl_3$

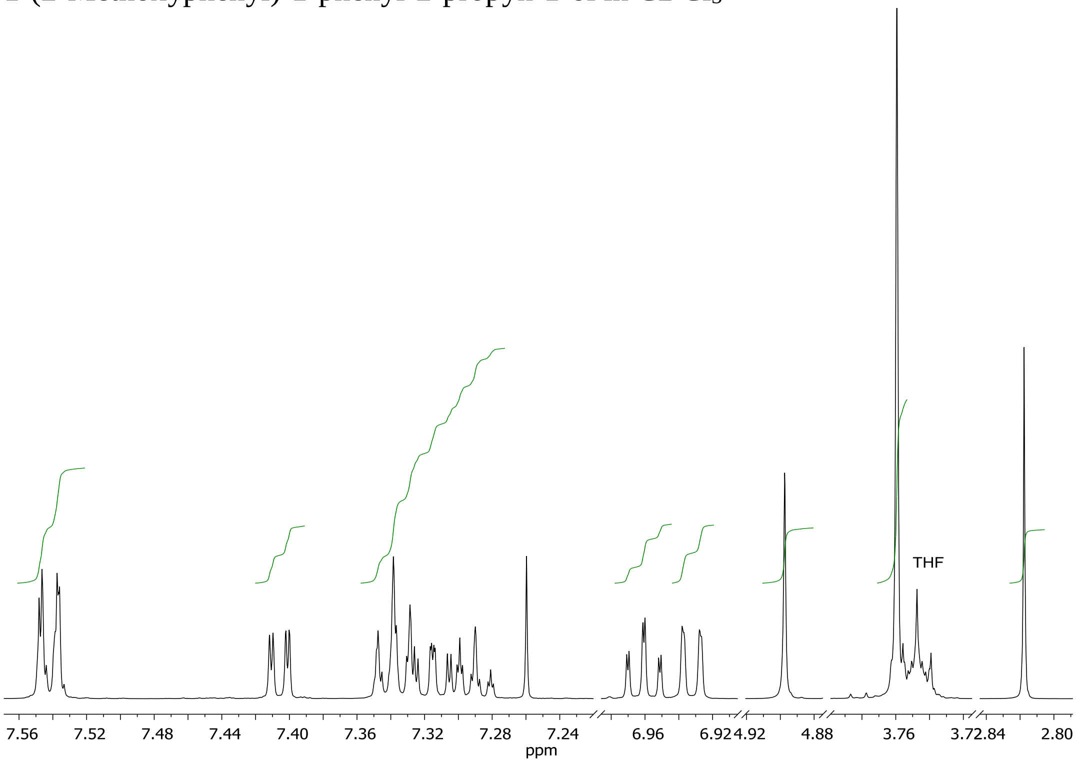

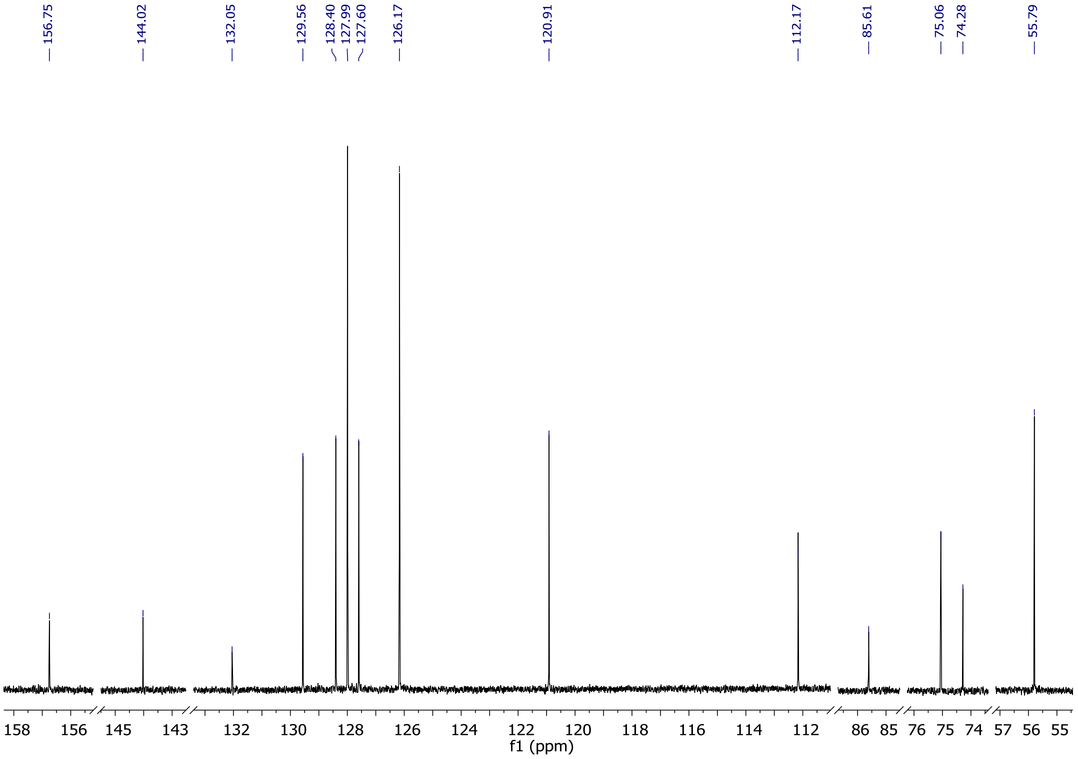

3-(2-Methoxyphenyl)-3-phenyl-3*H*-benzo[*f*]chromene in $CDCl_3$

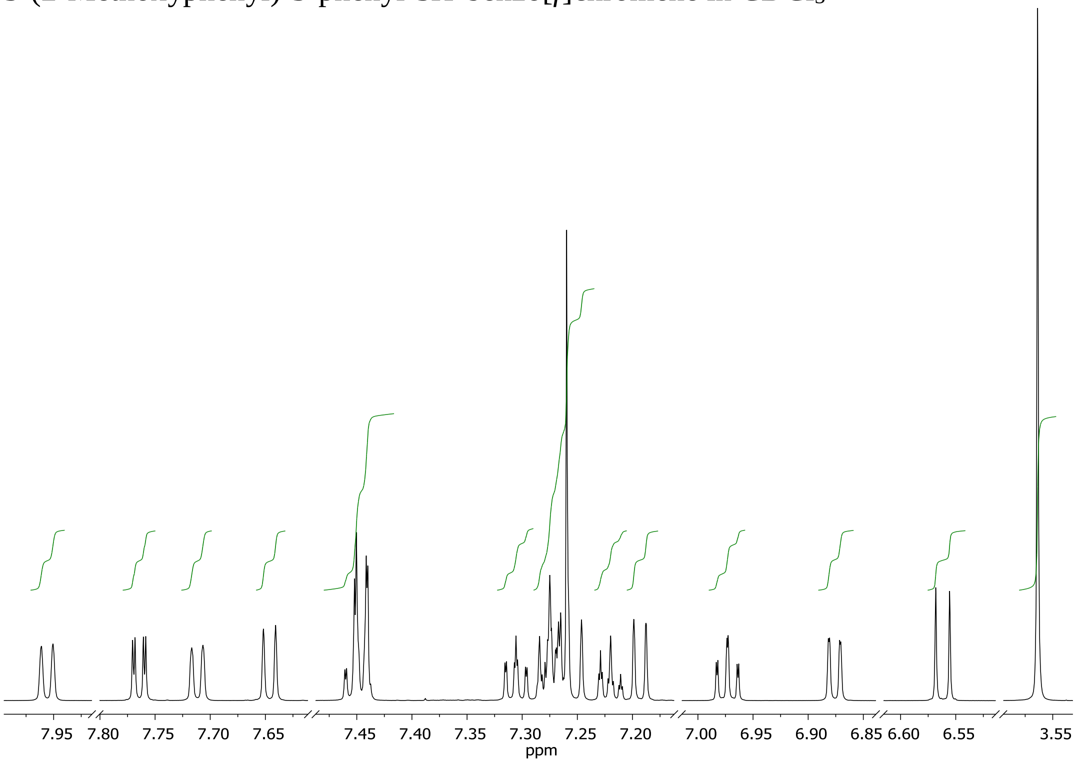

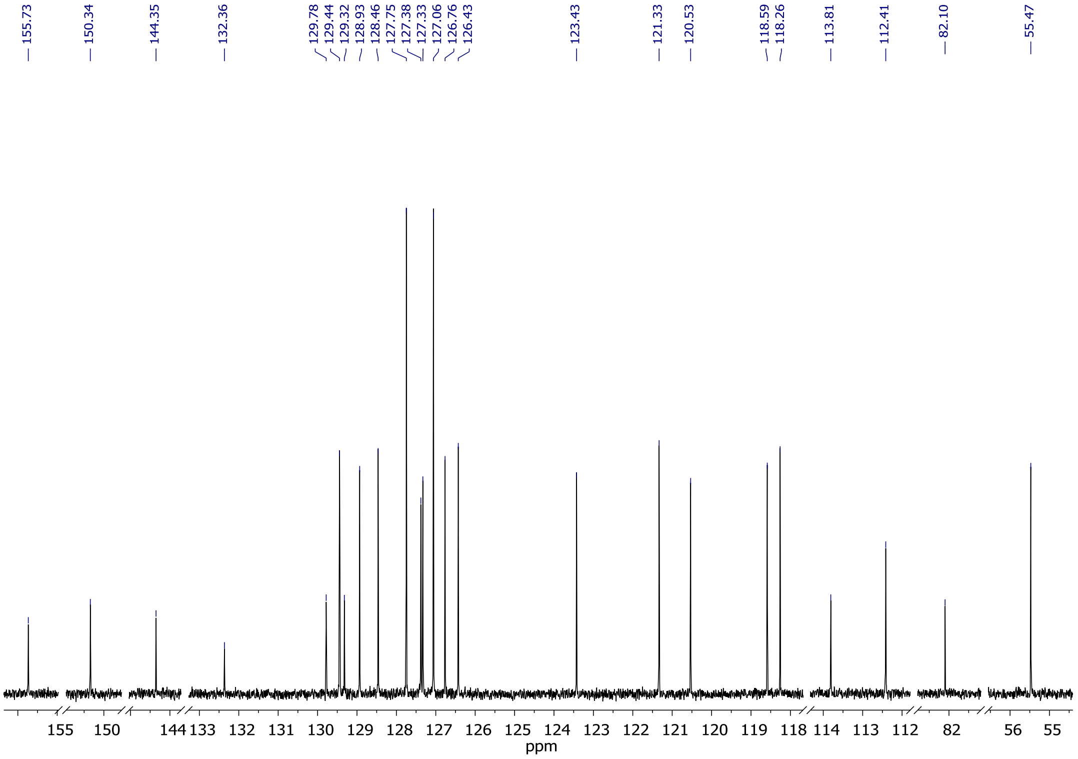

Figure S1. UV-vis molar extinction spectrum of **2** in cyclohexane.

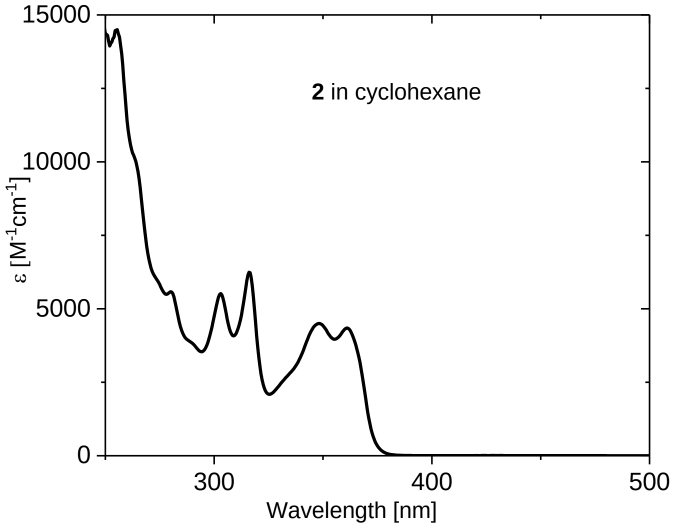

Figure S2. Colored species are generated upon UV irradiation at 365 nm (period 1 min, 4.1 mW/cm[2]) of **2** in cyclohexane (A(365) = 0.39) at 21 °C (A) Transient absorption UV-vis spectra determined at delays related to the moment of UV irradiation cessation (B) Kinetics trace probed at 421 nm can be fitted with a biexponential function, the short time-constant τ_1=17.5 min corresponds to **TC2** population, while the long component (τ_2 = 16 h) describes the **TT** population decay.

(A)

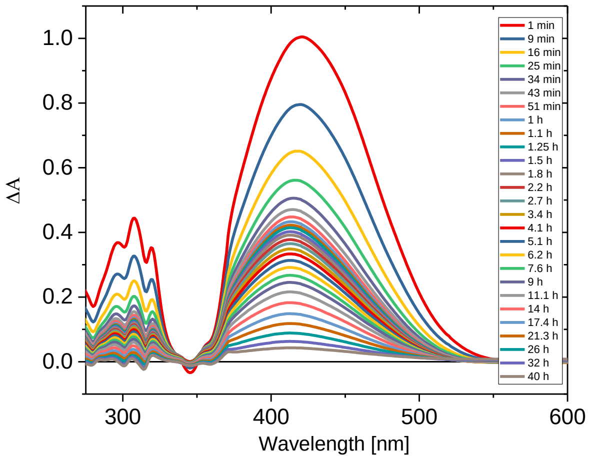

(B)

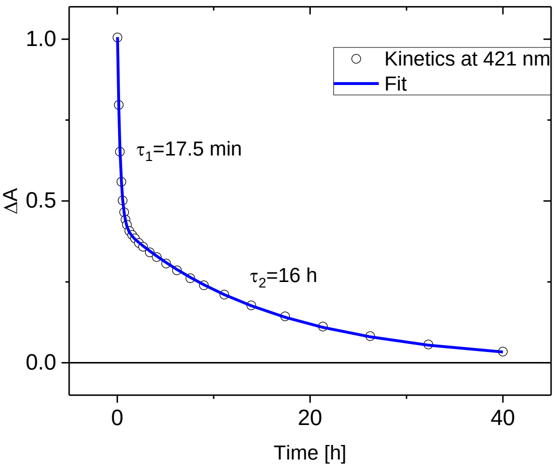

Table S1. Vertical excitation energy, ΔE^{VE} (in eV) and λ_{abs} (in nm), oscillator strength, *f*, and dipole moment, μ_e (in Debye), of the lowest excited singlet states calculated with the CC2/aug-cc-pVDZ method for the ground state equilibrium forms optimized at the MP2/cc-pVDZ theory level. Dipole moment of the ground-state, μ_g (in Debye, MP2/cc-pVDZ).

S_0 form		ΔE^{VE}	λ_{abs}	f	μ_e
**colorless CF forms**					
**CF$_a$** 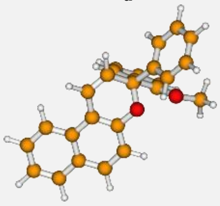	S_0	0.056 [a]			μ_g = 1.8
	$S_0 \rightarrow S_1(\pi\pi^*)$	**3.68**	**337**	**0.077**	2.5
	$S_0 \rightarrow S_2(\pi\pi^*)$	**4.24**	**293**	**0.082**	1.6
	$S_0 \rightarrow S_3(n\pi^*)$	4.55	273	0.004	4.9
	$S_0 \rightarrow S_4(n\pi^*)$	**4.70**	264	**0.073**	3.9
	$S_0 \rightarrow S_5(\pi\pi^*)$	4.79	**259**	0.004	8.8
	$S_0 \rightarrow S_6(\pi\pi^*)$	**4.84**	**256**	**0.085**	13.0
**CF$_b$** 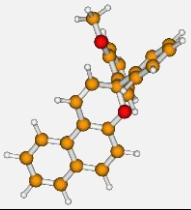	S_0	0.064 [a]			μ_g = 2.6
	$S_0 \rightarrow S_1(\pi\pi^*)$	**3.70**	**335**	**0.075**	4.1
	$S_0 \rightarrow S_2(\pi\pi^*)$	**4.22**	**294**	**0.044**	2.5
	$S_0 \rightarrow S_3(\pi\pi^*)$	4.59	270	**0.003**	3.7
	$S_0 \rightarrow S_4(\pi\pi^*)$	**4.73**	**262**	**0.018**	5.5
	$S_0 \rightarrow S_5(\pi\pi^*)$	**4.79**	**259**	**0.041**	3.7
	$S_0 \rightarrow S_6(n\pi^*)$	4.91	253	0.006	6.4
**CF$_c$** 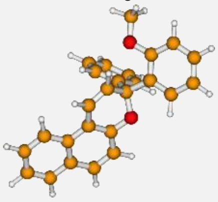	S_0	0.0			μ_g = 2.4
	$S_0 \rightarrow S_1(\pi\pi^*)$	**3.71**	**335**	**0.079**	3.2
	$S_0 \rightarrow S_2(\pi\pi^*)$	**4.24**	**293**	**0.078**	2.1
	$S_0 \rightarrow S_3(n\pi^*)$	4.60	270	0.005	4.1
	$S_0 \rightarrow S_4(\pi\pi^*)$	**4.77**	**260**	**0.098**	4.5
	$S_0 \rightarrow S_5(n\pi^*)$	4.80	259	0.005	7.8
	$S_0 \rightarrow S_6(\pi\pi^*)$	**4.95**	251	**0.039**	2.5
**CF$_d$** 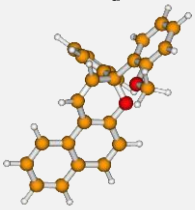	S_0	0.113 [a]			μ_g = 1.6
	$S_0 \rightarrow S_1(\pi\pi^*)$	**3.66**	**339**	**0.071**	3.0
	$S_0 \rightarrow S_2(n\pi^*)$	**4.23**	**293**	**0.047**	1.5
	$S_0 \rightarrow S_3(n\pi^*)$	4.58	271	0.003	4.4
	$S_0 \rightarrow S_4(\pi\pi^*)$	**4.66**	**266**	**0.005**	6.8
	$S_0 \rightarrow S_5(\pi\pi^*)$	**4.76**	**261**	**0.089**	2.5
	$S_0 \rightarrow S_6(n\pi^*)$	4.84	256	0.001	5.7
**colored TC forms**					
**CTC$_a$** 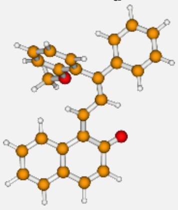	S_0	0.626 [a]			μ_g = 3.5
	$S_0 \rightarrow S_1(\pi\pi^*)$	**2.72**	**456**	**0.034**	2.1
	$S_0 \rightarrow S_2(\pi\pi^*)$	**2.94**	**422**	**0.659**	6.3
	$S_0 \rightarrow S_3(\pi\pi^*)$	**3.53**	**352**	**0.041**	8.5
	$S_0 \rightarrow S_4(\pi\pi^*)$	**3.74**	**332**	**0.061**	12.3
	$S_0 \rightarrow S_5(\pi\pi^*)$	**4.15**	**299**	**0.200**	6.6
	$S_0 \rightarrow S_6(\pi\pi^*)$	**4.34**	**286**	**0.045**	3.9
**CTC$_b$** 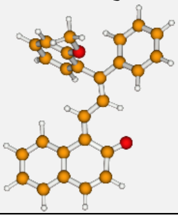	S_0	0.610 [a]			μ_g = 4.1
	$S_0 \rightarrow S_1(\pi\pi^*)$	**2.72**	**456**	**0.037**	1.4
	$S_0 \rightarrow S_2(\pi\pi^*)$	**2.97**	**418**	**0.718**	7.3
	$S_0 \rightarrow S_3(\pi\pi^*)$	**3.53**	**352**	**0.082**	8.3
	$S_0 \rightarrow S_4(\pi\pi^*)$	**3.82**	**325**	**0.033**	16.9
	$S_0 \rightarrow S_5(\pi\pi^*)$	**4.16**	**298**	**0.212**	7.3
	$S_0 \rightarrow S_6(n\pi^*)$	4.34	286	0.003	11.4

**TTC$_c$**	S_0	0.655 [a]			μ_g = 3.1
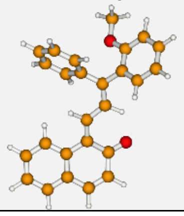	$S_0 \to S_1(\pi\pi^*)$	**2.74**	**453**	**0.028**	0.9
	$S_0 \to S_2(\pi\pi^*)$	**2.98**	**416**	**0.712**	7.6
	$S_0 \to S_3(\pi\pi^*)$	**3.55**	**350**	**0.082**	6.3
	$S_0 \to S_4(\pi\pi^*)$	**3.79**	**327**	**0.025**	14.9
	$S_0 \to S_5(\pi\pi^*)$	**4.16**	**298**	**0.069**	8.5
**TTC$_d$**	S_0	0.670 [a]			μ_g = 2.2
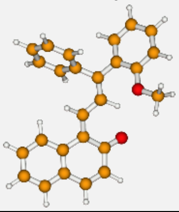	$S_0 \to S_1(\pi\pi^*)$	**2.70**	**460**	**0.095**	1.8
	$S_0 \to S_2(\pi\pi^*)$	**2.88**	**431**	**0.639**	6.0
	$S_0 \to S_3(\pi\pi^*)$	**3.53**	**352**	**0.074**	7.0
	$S_0 \to S_4(\pi\pi^*)$	**3.80**	**327**	**0.058**	10.7
	$S_0 \to S_5(\pi\pi^*)$	**4.15**	**299**	**0.111**	6.3
	$S_0 \to S_6(\pi\pi^*)$	**4.32**	**287**	**0.136**	3.1
**colored TT forms**					
**CTT$_a$**	S_0	0.595 [a]			μ_g = 2.1
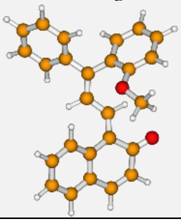	$S_0 \to S_1(\pi\pi^*)$	**2.87**	**432**	**0.156**	1.1
	$S_0 \to S_2(\pi\pi^*)$	**3.04**	**408**	**0.392**	6.0
	$S_0 \to S_3(\pi\pi^*)$	**3.60**	**345**	**0.069**	2.8
	$S_0 \to S_4(\pi\pi^*)$	**4.72**	**263**	**0.052**	2.8
	$S_0 \to S_5(\pi\pi^*)$	**4.21**	**295**	**0.096**	5.3
	$S_0 \to S_5(\pi\pi^*)$	**4.40**	**282**	**0.154**	3.6
**CTT$_b$**	S_0	0.650 [a]			μ_g = 3.0
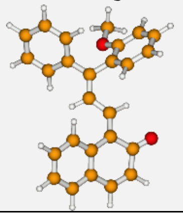	$S_0 \to S_1(\pi\pi^*)$	**2.81**	**442**	**0.118**	1.2
	$S_0 \to S_2(\pi\pi^*)$	**3.09**	**402**	**0.589**	7.3
	$S_0 \to S_3(\pi\pi^*)$	**3.64**	**341**	**0.054**	5.4
	$S_0 \to S_4(\pi\pi^*)$	**3.83**	**324**	**0.024**	14.4
	$S_0 \to S_5(\pi\pi^*)$	**4.22**	**294**	**0.133**	8.0
	$S_0 \to S_5(\pi\pi^*)$	**4.39**	**283**	**0.149**	3.9
**TTT$_c$**	S_0	0.676 [a]			μ_g = 4.1
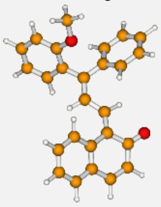	$S_0 \to S_1(\pi\pi^*)$	**2.83**	**439**	**0.109**	1.4
	$S_0 \to S_2(\pi\pi^*)$	**3.10**	**400**	**0.585**	8.2
	$S_0 \to S_3(\pi\pi^*)$	**3.65**	**340**	**0.046**	7.2
	$S_0 \to S_4(\pi\pi^*)$	**3.92**	**324**	**0.042**	16.9
	$S_0 \to S_5(\pi\pi^*)$	**4.23**	**293**	**0.133**	8.1
	$S_0 \to S_6(\pi\pi^*)$	**4.42**	**281**	**0.135**	4.4
**TTT$_d$**	S_0	0.694 [a]			μ_g = 4.4
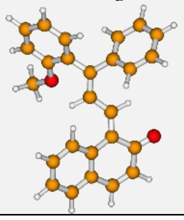	$S_0 \to S_1(\pi\pi^*)$	**2.80**	**443**	**0.185**	2.5
	$S_0 \to S_2(\pi\pi^*)$	**3.01**	**412**	**0.500**	6.9
	$S_0 \to S_3(\pi\pi^*)$	**3.64**	**341**	**0.032**	8.4
	$S_0 \to S_4(\pi\pi^*)$	**3.93**	**316**	**0.053**	13.5
	$S_0 \to S_5(\pi\pi^*)$	**4.21**	**295**	**0.160**	7.9
	$S_0 \to S_6(\pi\pi^*)$	**4.32**	**287**	**0.110**	5.8

Table S2. Comparison of geometric parameters (bond lengths, in Å, dihedral angles), adiabatic energies (E^a, in eV) of the excited state (S_1) and ground state (S_0) geometries of the species involved in photoreaction of **2** optimized respectively at the ADC(2)/cc-pVDZ and MP2/cc-pVDZ levels of theory, respectively. Here σ and τ denote C_{14}=C_1−C_2=C_3 and C_5−C_{13}−C_{14}=C_1 dihedral angles, respectively.

S_0- or S_1-State Form	R_1 C_3−O_4 [Å]	θ_1 C_{13}−C_{14}=C_1−C_2 [°]	θ_2 C_1−C_2=C_3−$C_{1'}$ [°]	σ [°]	τ [°]	R_2 C_{13}−O_4 [Å]	R_D C_1−C_{14} [Å]	R_S C_1−C_2 [Å]	R_E C_2−C_3 [Å]	E^a [eV]
S_0^{CFa}	1.454	19.7	81.7	3.0	172.0	1.368	1.460	1.360	1.516	0.056
S_0^{INTa}	2.835	17.6	6.0	43.0	154.8	1.240	1.389	1.446	1.380	0.844
S_0^{CTCa}	4.196	5.6	-4.3	172.9	159.4	1.239	1.384	1.435	1.382	0.626
S_1^{CTCa}	3.953	22.8	-12.7	-177.2	174.1	1.401	1.475	1.370	1.434	2.210
S_1^{TWa}	4.647	92.0	-7.0	175.5	178.3	1.261	1.474	1.366	1.437	1.944
S_1^{BPa}	4.298	61.0	-63.9	179.9	176.3	1.264	1.477	1.346	1.491	1.965
S_0^{CFb}	1.458	17.9	84.6	4.3	170.6	1.373	1.460	1.358	1.520	0.064
S_0^{INTb}	2.855	22.2	3.0	48.8	151.1	1.237	1.384	1.447	1.375	0.858
S_0^{CTCb}	4.175	3.0	-9.6	176.0	159.2	1.239	1.386	1.437	1.382	0.610
S_1^{CTCb}	3.914	20.1	-15.9	-174.8	174.1	1.401	1.475	1.376	1.431	2.187
S_0^{TWb}	4.521	87.4	-11.7	-179.9	178.7	1.261	1.474	1.371	1.436	2.004
S_1^{BPb}	4.311	66.9	-61.8	-176.4	175.7	1.265	1.482	1.343	1.488	2.106
S_0^{CFc}	1.458	19.6	80.4	3.1	172.2	1.371	1.461	1.359	1.514	0.000
S_1^{INTc}	2.769	17.7	8.2	47.3	150.9	1.239	1.387	1.449	1.376	0.906
S_0^{TTCc}	4.163	-1.7	8.5	-173.4	-159.6	1.239	1.385	1.439	1.383	0.655
S_1^{TTCc}	3.910	-22.9	16.9	174.0	-174.1	1.398	1.478	1.372	1.439	2.237
S_1^{TWc}	4.350	-80.5	18.4	174.4	-176.1	1.263	1.476	1.365	1.446	2.023
S_1^{BPc} collapses to S_1^{TWc}										
S_0^{CFd}	1.459	18.8	77.9	6.2	170.2	1.370	1.461	1.358	1.517	0.113
S_1^{INTd}	2.774	17.0	8.6	46.0	151.7	1.239	1.389	1.444	1.380	0.938
S_0^{TTCd}	4.171	2.2	-8.6	173.7	160.0	1.240	1.387	1.436	1.385	0.670
S_1^{TTCd}	3.995	25.7	-16.9	-177.2	175.7	1.402	1.482	1.369	1.443	2.166
S_1^{TWd}	4.496	87.7	-17.8	-176.6	178.4	1.264	1.477	1.363	1.452	1.929
S_1^{BPd}	4.345	57.9	-67.4	176.1	176.0	1.267	1.486	1.342	1.500	2.100

Table S3. Comparison of geometric parameters (bond lengths, in Å, dihedral angles), adiabatic energies (E^a, in eV) of the excited state (S_1) and ground state (S_0) geometries of the species involved in photoreaction of methyl derivative (3-(2-methylphenyl)-3-phenyl-3*H*-benzo[*f*]chromene) optimized respectively at the ADC(2)/cc-pVDZ and MP2/cc-pVDZ levels of theory, respectively. Here σ and τ denote C_{14}=C_1–C_2=C_3 and C_5–C_{13}–C_{14}=C_1 dihedral angles, respectively.

S_0- or S_1-State Form	R_1 C_3–O_4 [Å]	θ_1 C_{13}–C_{14}=C_1–C_2 [°]	θ_2 C_1–C_2=C_3–$C_{1'}$ [°]	σ [°]	τ [°]	R_2 C_{13}–O_4 [Å]	R_D C_1–C_{14} [Å]	R_S C_1–C_2 [Å]	R_E C_2–C_3 [Å]	E_a [eV]
				**CFa**						
				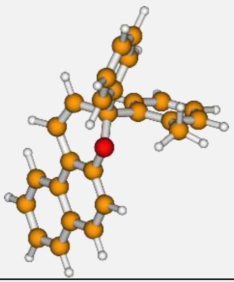						
S_0 ^{CFa}	1.459	20.0	82.4	2.4	172.1	1.372	1.460	1.360	1.517	0.00
S_0 ^{INTa}	2.832	18.1	5.6	44.5	153.8	1.239	1.387	1.447	1.380	0.779
S_0 ^{CTCa}	4.180	4.2	-5.9	172.6	160.3	1.239	1.385	1.437	1.382	0.554
S_1 ^{CTCa}	3.949	19.8	-13.6	-177.4	-175.2	1.402	1.475	1.373	1.432	2.130
S_1 ^{TWa}	4.497	88.9	-2.9	-177.5	178.5	1.261	1.474	1.370	1.435	1.954
S_1 ^{BPa}	4.274	61.1	-59.2	-177.9	176.5	1.265	1.482	1.343	1.492	1.971
				**CFb**						
				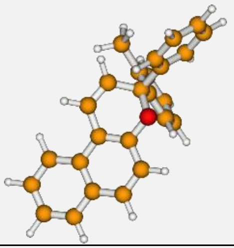						
S_0 ^{CFb}	1.461	19.0	81.8	5.4	169.1	1.372	1.459	1.359	1.519	0.095
S_0 ^{INTb}	2.843	20.2	4.0	44.0	158.0	2.843	1.387	1.446	1.379	0.857
S_0 ^{CTCb}	4.185	3.8	-7.8	175.9	159.1	1.239	1.385	1.438	1.383	0.538
S_1 ^{CTCb}	3.928	19.9	-13.9	-175.9	174.2	1.403	1.475	1.376	1.430	2.118
S_0 ^{TWb}	4.527	88.1	-9.4	179.9	179.1	1.261	1.474	1.371	1.436	1.907
S_1 ^{BPb}	--	--	--	--	--	--	--	--	--	------
				**CFc**						
				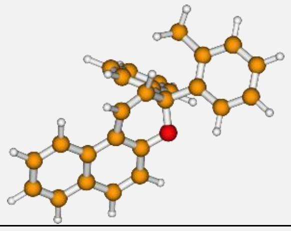						
S_0 ^{CFc}	1.462	20.6	78.1	3.3	171.3	1.370	1.460	1.360	1.515	0.031
S_0 ^{INTc}	2.766	17.5	9.5	45.8	151.1	1.241	1.389	1.445	1.378	0.835
S_0 ^{TTCc}	4.157	1.2	-8.3	173.5	158.3	1.239	1.385	1.440	1.383	0.605
S_1 ^{TTCc}	3.906	23.4	-15.9	-173.3	173.8	1.400	1.480	1.372	1.439	2.178
S_1 ^{TWc}	4.372	82.1	-15.6	-174.6	175.9	1.262	1.475	1.366	1.445	1.949
S_1 ^{BPc}	--	--	--	--	--	--	--	--	--	------

**CFd**										

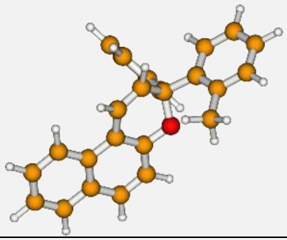

**S_0 CFd**	1.460	18.9	79.2	4.8	170.6	1.371	1.461	1.359	1.517	0.041
**S_0 INTd**	2.763	17.3	8.3	47.3	150.5	1.239	1.387	1.449	1.377	0.837
**S_0 TTCd**	4.166	2.9	-0.9	175.9	158.9	1.240	1.386	1.437	1.383	0.633
**S_1 TTCd**	3.973	16.9	-20.1	-178.4	175.5	1.400	1.477	1.373	1.437	2.172
**S_1 TWd**	4.382	83.5	-17.4	-173.8	177.5	1.264	1.475	1.368	1.441	1.947
**S_1 BPd**	4.317	61.7	-73.1	179.8	178.4	1.265	1.484	1.344	1.492	2.045

CF conversion yield, assignment to **TC** and **TT** populations and determination of molar extinction coefficients ε_{max} using time-resolved UV-vis and mid-IR absorption spectroscopies.

As we described in [1], simultaneous recording of the absorption changes in UV-vis and mid-IR spectral ranges of a photochromic system induced by UV light irradiation is a powerful method for spectroscopic insights into the photoreaction. Representative set of data recorded in the time window 0 –1600 s is shown below:

(A)

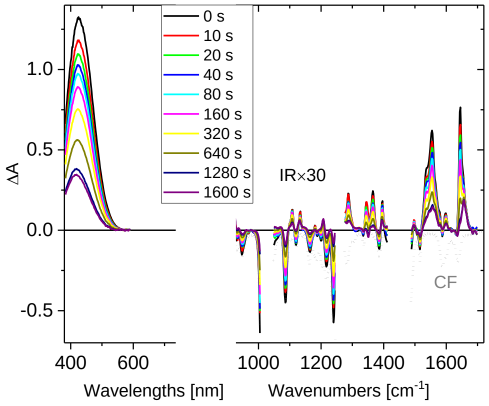

(B)

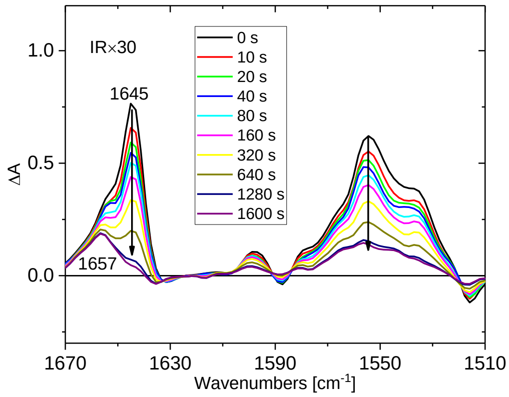

(C)

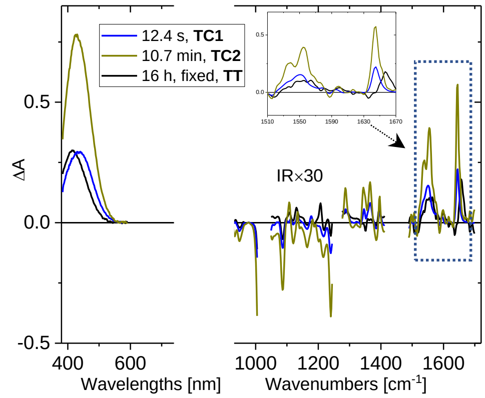

(D)

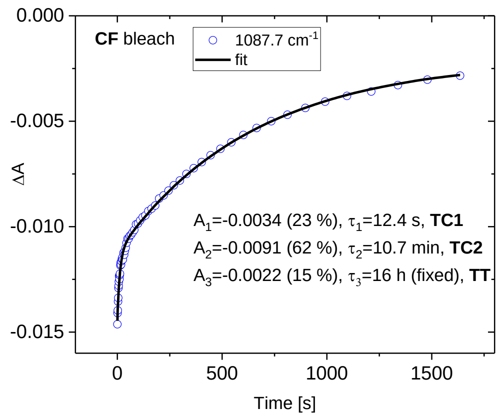

Figure S3. (A) Transient absorption UV-vis-IR spectra recorded after 60 s of UV irradiation (LED 365 nm, 2.4 mW) used for a solution of **2** in cyclohexane (2.69 $\times 10^{-3}$ M) at 26°C. The inverse stationary FTIR absorption spectrum of **CF** is also included. (B) Distinctive peaks of **TC** isomers appear at 1645 cm^{-1} and **TT** isomer at 1657 cm^{-1}. (C) Decay associated spectra $A_1(\lambda)$, $A_2(\lambda)$ and $A_3(\lambda)$ calculated by global analysis of data using a three exponential function $\Delta A(\lambda, t) = A_1(\lambda)\exp\left(-\frac{t}{\tau_1}\right) + A_2(\lambda)\exp\left(-\frac{t}{\tau_2}\right) + A_3(\lambda)\exp\left(-\frac{t}{\tau_3}\right)$. Revealed lifetimes 12.4 s (**TC1**) and 10.7 min (**TC2**), while the third component was set as a fixed value (16 h). The inset shows spectral details in 1510-1670 cm^{-1}. (D) **CF** bleach recovery kinetics at 1087.7 cm^{-1} occurs through thermal decay of **TC1**, **TC2** and **TT**. The fit is from global analysis (solid line). The relative path yield is estimated from the amplitude contribution $A_i/(A_1 + A_2 + A_3)$.

Figure S4A shows the transient spectra in the spectral ranges 380–580 nm and 1700–930 cm[1]. At time of 0 s, a mixture of **TC1**, **TC2**, **TT** (positive bands) and the bleached **CF** species (negative bands) are observed. The bleached **CF** band at 0s can be compared with the scaled stationary FTIR band of the fresh **CF** solution. The found scaling factor is 0.38, which means that UV irradiation **converted 38 %** of the initial **CF** population. Figure S4B shows that IR data can clearly distinct spectrally the C=O vibrational band of the **TC** isomer (1645 cm^{-1}) and **TT** isomer (1657 cm^{-1}), in agreement with the reported [1] values for the reference compound **1** (**TC** at 1644 cm^{-1} and **TT** at 1655 cm^{-1}). Figure S4C shows the decay associated spectra obtained by global analysis of recorded data, the inset shows clearly the spectral difference between **TC** and **TT** isomers, however, **TC1** and **TC2** show practically identical spectral signature. Thermal decays of **TC1** and **TC2** are characterized by time-constants 12.4 s and 10.7 min, respectively, which are shorter than those in Figure 2 (17.1 s and 17.5 min), due to the difference in temperature (26°C vs 21°C). Figure S4D presents the **CF** bleaching recovery, which can provide approximate information on relative **TC1**, **TC2** and **TT** contributions: 23 %, 62 % and 15 %. The highest isomer population is **TC2** with estimated concentration of $[\mathbf{TC2}]_0=0.63\times10^{-3}$ M on the basis of (1) **CF** conversion yield of 0.38 (2) 62 % **TC2** contribution in the **CF** bleached signal. Using the absorbance maximum A(TC2) in the visible range (see Fig. S4C – left side) one can calculate the respective molar extinction coefficients ε_{max} according to the formula:

$$\varepsilon_{max} = \frac{A(TC2)}{[TC2]_0\, L\, f} \qquad [1]$$

where $A(\boldsymbol{TC2})$ is the respective isomer absorbance at time 0 s, A(**TC2**)=0.778 (Figure S4C),

$[\boldsymbol{TC2}]_0$ is the initial molar isomer concentration, $[\mathbf{TC2}]_0=0.63\times10^{-3}$ M

L is the optical length of solution (0.063 cm)

f is the angle correction factor (1.05) since UV-vis probe beam passes through a slightly longer solution layer in comparison to that of mid-IR beam. The angle of between IR and UV-vis beams is about 17°.

The obtained molar extinction coefficients for **TC2** is $\varepsilon_{max} \approx$ 18700 $M^{-1}cm^{-1}$, which is in excellent agreement with the one reported for **TC** isomer (18100 $M^{-1}cm^{-1}$) photogenerated from reference compound **1** [2].

The method applied for **TC2**, can work also for **TC1** revealing $\varepsilon_{max} \approx$ 18900 $M^{-1}cm^{-1}$, while for **TT** $\varepsilon_{max} \approx$ 29500 $M^{-1}cm^{-1}$, however their accuracies are certainly lower due to smaller signal contribution in the UV-vis-IR transient absorption spectra.

Figure S4. Thermal decoloration **TC↔INT→CF** for four starting **TC** geometries: **CTC$_a$, CTC$_b$, TTC$_c$** and **TTC$_d$.**

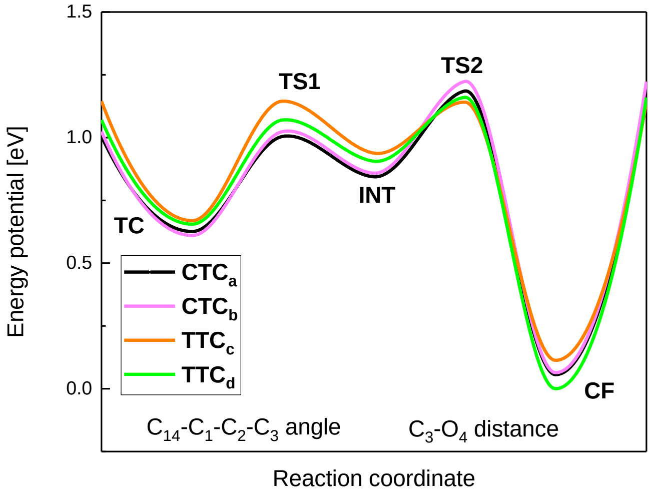

References:

1 Brazevic, S., Nizinski, S., Szabla, R., Rode, M. F. & Burdzinski, G. Photochromic reaction in 3*H*-naphthopyrans studied by vibrational spectroscopy and quantum chemical calculations. *Phys. Chem. Chem. Phys.* **21**, 11861-11870, doi:https://doi.org/10.1039/C9CP01451A (2019).

2 Zhao, W. & Carreira, E. M. Oligothiophene-linked bisnaphthopyrans: sequential and temperature-dependent photochromism. *Chem. Eur. J.* **13**, 2671-2685 (2007).
